# Therapeutic potential of boric acid as a local drug delivery agent in periodontitis: a comprehensive systematic review and meta-analysis

**DOI:** 10.1186/s12903-025-05445-0

**Published:** 2025-01-17

**Authors:** Reham Abdel-Fatah, Ghada A. Elhusseiny, Wafaa Saleh

**Affiliations:** Oral Medicine, Periodontology, Diagnosis and Oral Radiology Department, Faculty of Dentistry, Mansoura University, Mansoura City, 33516 Egypt

**Keywords:** Boric acid, Periodontal diseases, Local drug delivery, Systematic review, Meta-analysis

## Abstract

**Objective:**

This systematic review and meta-analysis aim to evaluate the therapeutic potential of boric acid as a local drug delivery agent in the treatment of periodontitis.

**Methods:**

Following PRISMA guidelines, we registered a comprehensive protocol with PROSPERO. By employing PICOS criteria, we evaluated randomized controlled trials assessing the effects of subgingival boric acid application alongside non-surgical periodontal therapy in treatment of periodontitis. Studies were systematically searched across multiple databases, with establishment of the eligibility criteria. Data extraction and risk of bias assessment were conducted independently by reviewers.

**Results:**

Among 1,640 records screened, 6 studies met the inclusion criteria, comprising 281 participants aged 18–65 years. At 1-month, boric acid demonstrated significant improvements in probing pocket depth (PPD), but insignificant differences were observed in clinical attachment level (CAL), and gingival index (GI). However, at 3 and 6 months, we found significant reductions in PPD while at 6 months, a significant increase in CAL gain were observed favoring boric acid. No significant changes in GI were noted at any follow-up duration.

**Conclusion:**

Boric acid adjunctive therapy in non-surgical periodontal treatment shows promising efficacy in improving clinical parameters, particularly PPD and CAL, over time. While early outcomes may not show significance, sustained benefits are evident at longer follow-up periods. These findings underscore the potential of boric acid as a valuable addition to periodontal therapy, demanding further investigation to reveal its precise mechanisms and optimize clinical application.

**Supplementary Information:**

The online version contains supplementary material available at 10.1186/s12903-025-05445-0.

## Introduction

Periodontal diseases represent a group of inflammatory conditions affecting the supporting structures of the teeth, including the gingiva, alveolar bone, and periodontal ligament. Periodontitis involves the progressive loss of connective tissue attachment and bone around the teeth. Untreated periodontal diseases can lead to tooth mobility and eventual tooth loss, significantly impacting oral health and quality of life. It typically arises as a result of the body’s immune system reacting to pathogenic bacteria found in dental plaque, resulting in a cascade of inflammatory processes [1, 2].

The treatment of periodontitis requires a comprehensive approach, including non-surgical techniques as scaling and root planing (SRP), as well as more advanced surgical procedures such as flap surgery and bone grafting [3–5]. Despite advancements in treatment modalities of periodontitis, achieving optimal outcomes can be challenging, and researchers continue to explore novel therapeutic strategies to enhance the effectiveness of periodontal interventions [5, 6]. 

The introduction of local drug delivery marks a significant advancement in periodontitis treatment. While mechanical debridement is crucial for reducing overall pathogen load, it faces limitations in addressing subgingival infections in inaccessible areas [7]. To overcome this, a combined approach of mechanical debridement and antimicrobial therapy, involving topical antibiotics and antiseptics, has been proposed. Local drug delivery proves more targeted and effective compared to systemic antibiotics, which can lead to superinfections and increased resistance [8]. Drawbacks of topical antibiotics, such as re-infection risks and poor success rates emphasize the need for a nuanced, localized treatment strategy [9]. Various antimicrobial agents, anti-inflammatory drugs, and host modulatory agents have been investigated in the context of local drug delivery for periodontal diseases [7, 10, 11]. 

Boric acid plays a significant role in the treatment of periodontitis due to its low toxicity and notable antiseptic, antifungal, and antiviral properties. Recent studies, such as one conducted by Luan et al., highlight the effectiveness of boric acid in treating experimentally induced periodontitis in rats. This compound demonstrates both anti-inflammatory and antibacterial characteristics, reducing bone loss and inflammatory infiltrate formation [12]. Another investigation reveals that systemic administration of boric acid in periodontitis, induced by ligature in diabetic rats, mitigates alveolar bone loss by increasing osteoblast count and regulating the oxidant-antioxidant balance [13]. Overall, boric acid has demonstrated antimicrobial and anti-inflammatory properties. In recent years, researchers have explored its potential application as a local drug delivery agent in the treatment of periodontitis. Preliminary studies suggest that boric acid may exhibit promising effects in controlling periodontal pathogens and mitigating inflammatory processes within the periodontal tissues [14, 15]. 

The aim of this systematic review and meta-analysis is to comprehensively evaluate the efficacy of boric acid as a local drug delivery agent in the treatment of periodontitis, specifically comparing its effects to a placebo. By synthesizing existing evidence from relevant studies, we seek to provide a thorough understanding of the potential benefits and limitations of boric acid in the treatment of periodontitis. This analysis aims to inform clinicians and researchers about the current state of knowledge regarding boric acid’s role in periodontal care, with implications for future research directions and clinical applications.

## Methods

### Protocol registration

The current systematic review adhered to a comprehensive protocol in accordance with the guidelines outlined in Preferred Reporting Items for Systematic Reviews and Meta-Analyses (PRISMA) [16]. The protocol detailing this systematic review has been officially registered with PROSPERO (International Prospective Register of Systematic Reviews) under the identification code CRD42024518452.

### Study PICOS questions

The formulated PICOS (Population, Intervention, Comparison, Outcomes, and Study Design) question can be expressed as follows:

#### Population

Individuals with overall systemic health who have been diagnosed with periodontitis.

#### Intervention

The intervention includes SRP combined with the localized application of boric acid gel in the treatment of periodontitis.

#### Comparison

The comparison periodontitis groups undergo SRP alongside the administration of a placebo.

#### Outcomes

The expected outcomes center on clinical changes following periodontal treatment. The primary emphasis is on the reduction in probing pocket depth (PPD) and clinical attachment loss (CAL). Secondary outcomes encompass reduction in the gingival index (GI).

#### Study design

The studies will include randomized controlled clinical trials framework, with follow-ups scheduled at 1 month, 3 months, and 6 months.

### Eligibility criteria

Inclusion in this systematic review was extended to human randomized clinical trials diagnosed with periodontitis with a minimum follow-up of one, three months or six months. These trials evaluated the efficacy of subgingival boric acid application as an adjunct to SRP in comparison to SRP with a placebo. Studies focusing on non-surgical periodontal treatment were eligible, while those involving surgical interventions were excluded. The inclusion criteria required that selected studies must report at least one of the specified outcomes, and language restrictions were not performed.

### Exclusion criteria

#### Studies were disqualified if they

- Included systemic antimicrobials as an intervention.
- Utilized local anti-infective therapy alone (monotherapy).
- Prolonged the time between SRP and local antimicrobial administration.
- Implemented any local or systemic antimicrobial treatment before or after baseline.
- Studies applied on gingivitis cases.

#### Additional exclusion criteria encompassed

- Studies involving patients with systemic diseases.
- Studies involving smokers, pregnant and lactating females.
- Abstracts of posters or presentations.
- Studies that did not report treatment outcomes of interest for the present review.
- Case reports and case series.

### Search and screening strategy

The search strategy involved utilizing Mesh terms, including boric acid, periodontitis, local drug, SRP, subgingival irrigation, along with related terms. Complementary keywords such as “local application,” or “topical application,” or” combined with terms like “Periodontal Pocket,” “Periodontal Disease,” were applied in the search.

A meticulous search was conducted across five electronic databases with the previously mentioned Mesh terms, namely Cochrane Central Register of Controlled Trials, MEDLINE, PubMed, EMBASE, and Web of Science. Grey literature searches, hand searches of journals, and expert consultations complemented the comprehensive approach.The search terms for each database were listed in supplementary file 1.

Two independent reviewers (GE &WS) conducted comprehensive electronic and manual searches in databases, including MEDLINE-PubMed, Embase, and Scopus, up to 10 February 2024, without language restrictions. Manual searches extended to relevant journals, and grey literature exploration involved consulting clinicaltrials.gov for ongoing or completed randomized clinical trials.

Independent reviewers (RA, GE, and WS) conducted a thorough screening of studies based on title and abstract relevance, adhering to predefined eligibility criteria. Any discrepancies were resolved through discussion, and inter-reviewer agreement was quantified using kappa scores.

### Data extraction

Templates were systematically employed to record general study information, including study design, periodontal status, follow-up periods, and participant demographics. Details on baseline and end-of-study outcomes and treatment protocols were meticulously extracted. Mean values and standard deviations for changes in periodontal parameters were recorded for subsequent analysis.

This review data was extracted in a Microsoft Excel spreadsheet by reviewer (RA&GE) and further revised by reviewer (W.S) and included the general characteristics of the included studies (Table 1), clinical characteristics of the included studies (Table 2), and the clinical parameters of the included studies at different time intervals (Table 3).Any disagreement among the reviewers was clarified by open discussion.

### Data analysis

The meta-analysis was performed using Reviewer Manager Software, version 5.3 (Copenhagen: The Nordic Cochrane Centre, The Cochrane Collaboration, 2014). The analysis of the clinical parameters including PPD, CAL, and GI was carried out using the means and standard deviations, and the weight of each study contribution. The level of significance considered (*p* ≤ 0.05), and the (CI = 95%). The fixed effect model used with low heterogeneity results (*p* ≥ 0.10, I² ≤ 50%) while random effect model used with high heterogeneity results (*p* < 0.10, I^2^ > 50%).

**Fig. 1:**
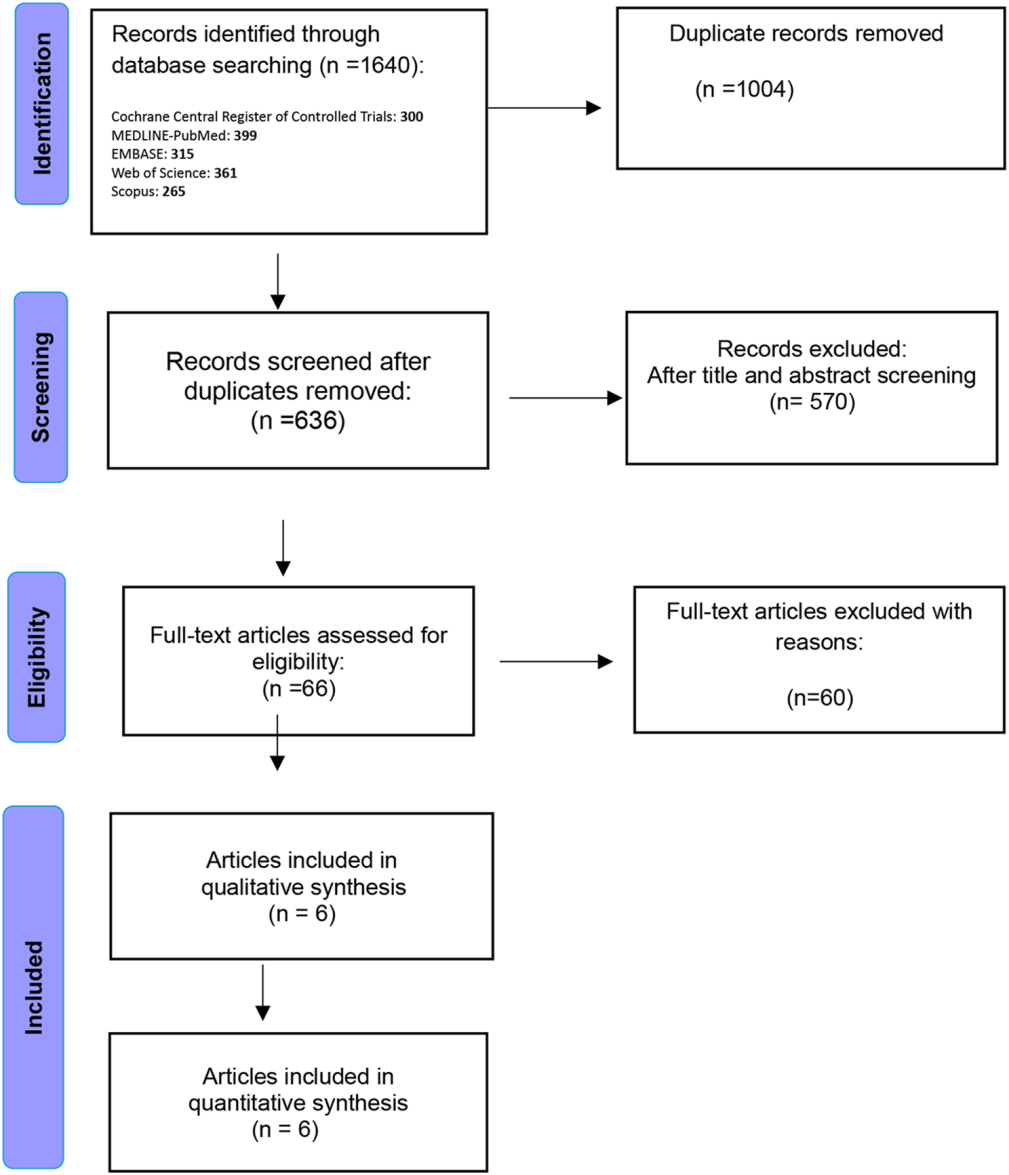
Prisma flow diagram of the included studies

**Fig. 2:**
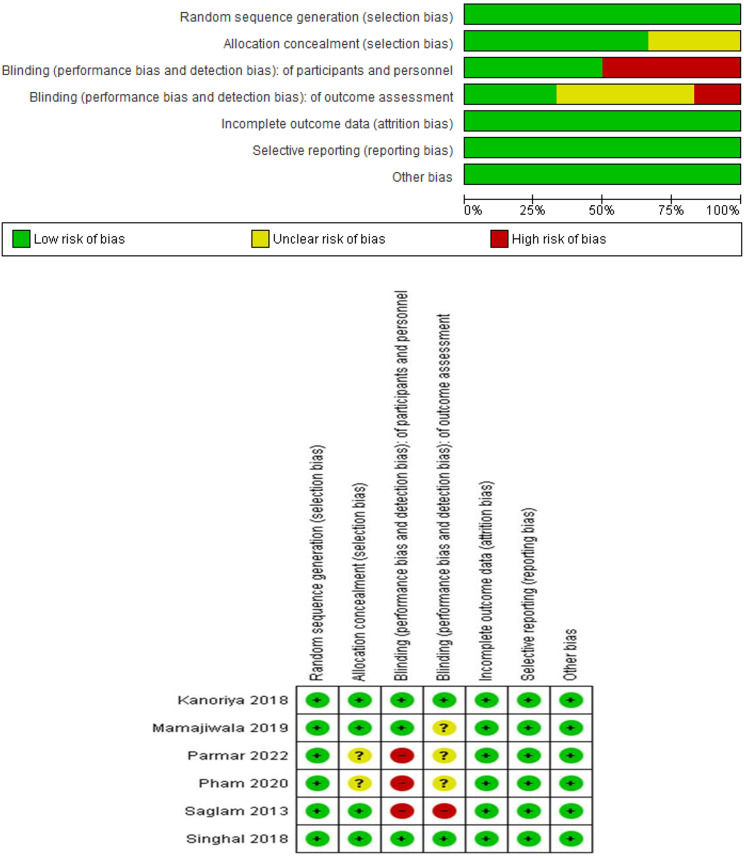
Risk of bias graph and summary

## Results

In our systematic review and meta-analysis, the search across various databases yielded a total of 1,640 records. Grey literature searches and hand searches yield no more eligible studies. After removing duplicate records (*n* = 1,004), 636 records underwent screening. Among these, 570 records were excluded as they did not meet the inclusion criteria. Following the title and abstract screening, 66 full-text articles were assessed for eligibility. Of these, 60 articles were excluded, and the reasons for exclusion were documented. Consequently, 6 studies met the inclusion criteria and were included in both qualitative and quantitative (meta-analysis synthesis) phases as shown in Fig. 1: Study Flowchart.

### Assessment of the risk of bias among the selected studies

The risk of bias in the studies included in this meta-analysis was evaluated using the Cochrane Collaboration’s two-part tool, which is a well-established framework for assessing the methodological quality and internal validity of individual trials.

The assessment was conducted independently by two reviewers (RA & GE), and any discrepancies were resolved through discussion with a third reviewer (WS). Employing the Cochrane Collaboration’s two-part tool for bias risk assessment [17], efforts were made to gauge bias across studies and identify papers with inherent methodological and design flaws. The factors examined for a low, high, or unclear risk of bias included: (1) random sequence generation, (2) allocation concealment, (3) blinding of participants/personnel, (4) incomplete outcome data, (5) selective reporting outcomes, and (6) other potential risks of bias. The level of bias was categorized as low risk if all criteria were met, moderate risk if one criterion was missing, and high risk if two or more criteria were absent. Of the included studies, the high risk of bias was detected in 3 studies. The most frequently observed issues were blinding. These findings are summarized in Fig. 2.

### Characteristics of included studies

The 6 studies selected for qualitative synthesis encompass various randomized clinical trials investigating the therapeutic potential of boric acid as a local drug delivery agent in treatment of periodontitis. All studies were conducted independently and published between 2013 and 2022. The total number of included participants across the studies is 281 and the age range of participants across the studies ranges from 18 to 65 years. All the included studies excluded patients with a history of tobacco use (Table 1).

**Table 1:** General characteristics of included studies

Study ID	Study design	No. of participants	Age range (in years)	Gender (Number of males and females)		smoking status
				Males	Females	
Saglam et al. 2013 [18]	Randomized clinical trial	30	32–63	15	15	Excluded
Singhal et al. 2018 [14]	Randomized clinical trial	64	30–35	34	30	Excluded
Kanoriya et al. 2018 [15]	Randomized clinical trial	39	25–55	NR	NR	Excluded
Mamajiwala et al. 2019 [20]	Randomize clinical trial	30	18–55	15	15	Excluded
Pham et al. 2020 [21]	Randomized clinical trial	36	29–65	18	18	Excluded
Parmar et al. 2022 [19]	Randomized clinical trial	82	≥ 30	37	45	Excluded

### Follow up

We included a varied approach to follow-up durations, containing short-term, mid-term, and long-term perspectives. Singhal et al. (2018) [14]and Kanoriya et al. (2018) [15] conducted follow-ups at both 3 and 6 months, providing valuable insights into sustained impacts. Similarly, Saglam et al. (2013) [18] and Parmar et al. (2022) [19] systematically assessed participants at 1 and 3 months. Mamajiwala et al. (2019) [20] chose a 6-month follow-up period, facilitating an extended evaluation of outcomes. Pham et al. (2020) [21] conducted follow-ups at 1 and 2 months. Table 2.

**Table 2:** Clinical characteristics of included studies

Study ID	Type of periodontal disease	Number of patients in each group		Form of Boric acid	Mode of treatment in study group	Mode of treatment in control group	Follow up duration	Main outcomes of the study
		Study group	Control group					
Saglam et al.2013 [18]	Chronic periodontitis	15	15	Boric acid solution	SRP + boric acid irrigation	SRP + chlorhexidine irrigation	1 & 3 months	Boric acid irrigation may act as an alternative therapy to chlorohexidine as it shows superior decrease in the GI, PPD and CAL gain.
Singhal et al. 2018 [14]	Chronic periodontitis	29	29	Boric acid gel	SRP + boric acid gel	SRP + placebo gel	3 & 6 months	Boric acid group showed a significant decrease of the PPD and significant CAL gain than the placebo gel
Kanoriya et al.2018 [15]	Chronic periodontitis	20	19	Boric acid gel	SRP + boric acid gel	SRP + placebo gel	3&6 months	The mean PPD reduction and mean CAL gain were greater in the boric acid group than the placebo group at 3 and 6 months.
Mamajiwala et al. 2019 [20]	Chronic periodontitis	15	15	Boric acid gel	SRP + boric acid gel	SRP + chlorhexidine gel	6 months	Boric acid gel and CHX gel both were equally effective in improving the clinical and microbiologic parameters in patients with chronic periodontitis
Pham et al. 2020 [21]	Chronic periodontitis	18	18	Boric acid solution	SRP + boric acid irrigation	SRP + Povidone iodine irrigation	1&2 months	Boric acid showed more significant reduction of PPD and CAL gain in moderately deep pockets than povidone iodine solution
Parmar et al.2022 [19]	Chronic periodontitis	41	41	Boric acid solution	SRP + boric acid irrigation	SRP + chlorohexidine irrigation	1& 3 months	Boric acid showed significant reduction of GI, PPD and CAL gain in comparison to chlorhexidine

CHX: chlorhexidine

**Table 3:** Comparison of the studies’ outcomes between baseline and follow up durations

Study ID	Study groups	Baseline parameters			Follow up parameters								
		PPD	CAL	GI	After 1 month			After 3 months			After 6 months		
					PPD	CAL	GI	PPD	CAL	GI	PPD	CAL	GI
Saglam et al.,2013 [18]	BA group	5.16 ± 0.36	4.30 ± 0.82	1.80 ± 0.07	3.09 ± 0.53	2.23 ± 0.87	1.15 ± 0.04	2.56 ± 0.15	1.99 ± 0.80	0.99 ± 0.04	NR	NR	NR
	Control group	5.23 ± 0.42	4.39 ± 0.71	1.84 ± 0.13	3.25 ± 0.53	2.41 ± 0.79	1.18 ± 0.08	2.68 ± 0.12	2.14 ± 0.66	0.99 ± 0.11			
Singhal et al. 2018 [14]	BA group	7.12 ± 1.20	8.20 ± 1.35	2.56 ± 0.28	NR	NR	NR	4.92 ± 1.15	6.16 ± 1.31	0.41 ± 0.12	3.56 ± 0.96	5.28 ± 1.59	0.39 ± 0.14
	Control group	7.21 ± 1.44	7.91 ± 1.34	2.53 ± 0.26				5.78 ± 1.34	6.95 ± 1.36	1.44 ± 0.18	4.86 ± 1.35	6.39 ± 1.19	0.73 ± 0.21
Kanoriya et al.2018 [15]	BA group	6.75 ± 0.85	5.70 ± 0.65	NR	NR	NR	NR	4.9 ± 0.64	4.25 ± 0.55	NR	3.6 ± 0.50	3.05 ± 0.60	NR
	Control group	6.94 ± 0.91	5.78 ± 1.08					6.05 ± 0.77	5.10 ± 0.87		5.05 ± 0.70	4.47 ± 0.61	
Mamajiwala et al. 2019 [20]	BA group	6.78 ± 1.48	2.15 ± 1.01	1.21 ± 0.22	NR	NR	NR	NR	NR	NR	4.35 ± 0.62	1.21 ± 0.89	0.74 ± 0.35
	Control group	7.21 ± 1.12	2.32 ± 0.98	1.34 ± 0.22							4.60 ± 0.89	1.32 ± 1.10	0.69 ± 0.38
	Control group	2.40 ± 1.27		1.78 ± 0.39	1.89 ± 0.87		1.03 ± 0.43						
Pham et al.2020 [21]	BA group	5.21 ± 0.41	5.77 ± 1.41	1.39 ± 0.36	3.52 ± 0.84	4.22 ± 1.37	0.76 ± 0.40	NR	NR	NR	NR	NR	NR
	Control group	5.23 ± 0.42	5.78 ± 1.22	1.42 ± 0.33	3.73 ± 0.87	4.29 ± 1.46	1.05 ± 0.37						
Parmar et al. 2022 [19]	BA group	5.44 ± 0.12	2.34 ± 0.98	1.75 ± 0.12	3.87 ± 0.81	1.25 ± 0.67	1.06 ± 0.69	1.69 ± 0.5	0.65 ± 0.5	1.03 ± 0.34	NR	NR	NR
	Control group	5.29 ± 0.13	2.46 ± 0.87	1.78 ± 0.14	4.11 ± 0.61	1.51 ± 0.86	1.1 ± 0.47	1.87 ± 1.2	0.7 ± 0.6	1.27 ± 0.44			

BA: Boric acid

PPD: probing pocket depth

CAL: Clinical attachment level

GI: Gingival index

NR: Not reported

### Interventions

The mode of intervention in the study groups across the included studies in the systematic review entails the application of boric acid in two forms and methodologies. Specifically, Singhal et al. (2018) [14], Kanoriya et al. (2018) [15], and Mamajiwala et al. (2019) [20] implemented SRP along with the application of boric acid gel in their respective study groups. On the other hand, Parmar et al. (2022) [19], Pham et al. (2020) [21], and Saglam et al. (2013) [18] utilized SRP coupled with boric acid irrigation in their study groups. The treatment modalities for the control groups involved SRP in addition to Placebo gel, chlorhexidine gel, solution, or Povidone iodine irrigation. Table 2.

### Comparisons and clinical outcomes

Upon comparing the one-month follow-up data to baseline readings across all studies included in this systematic review, distinct variations in periodontal parameters emerge. In the study by Saglam et al. (2013) [18], the boric acid group demonstrated significant improvements in PPD, CAL, and GI at one month, while the control group exhibited similar favorable outcomes. Similarly, Parmar et al. (2022) [19] reported favorable reductions in PPD, CAL, and GI in the boric acid group, contrasting with lesser improvements in the control group. Pham et al. (2020) [21] showcased positive trends in the boric acid group, particularly in PPD and GI, when compared to the control group.

A comparative analysis of clinical outcomes at 3 months compared to baseline across multiple studies showed that the pooled data suggests a potential positive effect of boric acid on periodontal health. Four studies Singhal et al. (2018) [14], Saglam et al. (2013) [18], Kanoriya et al. (2018) [15], and Parmar et al. (2022) [19]reported that the boric acid group exhibited significant improvements in PPD and CAL compared to the control group. Saglam et al. (2013) [18] and Kanoriya et al. (2018) [15] also explored the effects of boric acid, with varied baseline parameters.

At 6 months follow up, three studies [14, 15, 20] reported a significant improvement of the PPD and CAL while only two studies [14, 20] measured the changes in the GI at 6 months in comparison to baseline.

### Meta-analysis

#### Probing pocket depth

**Fig. 3:**
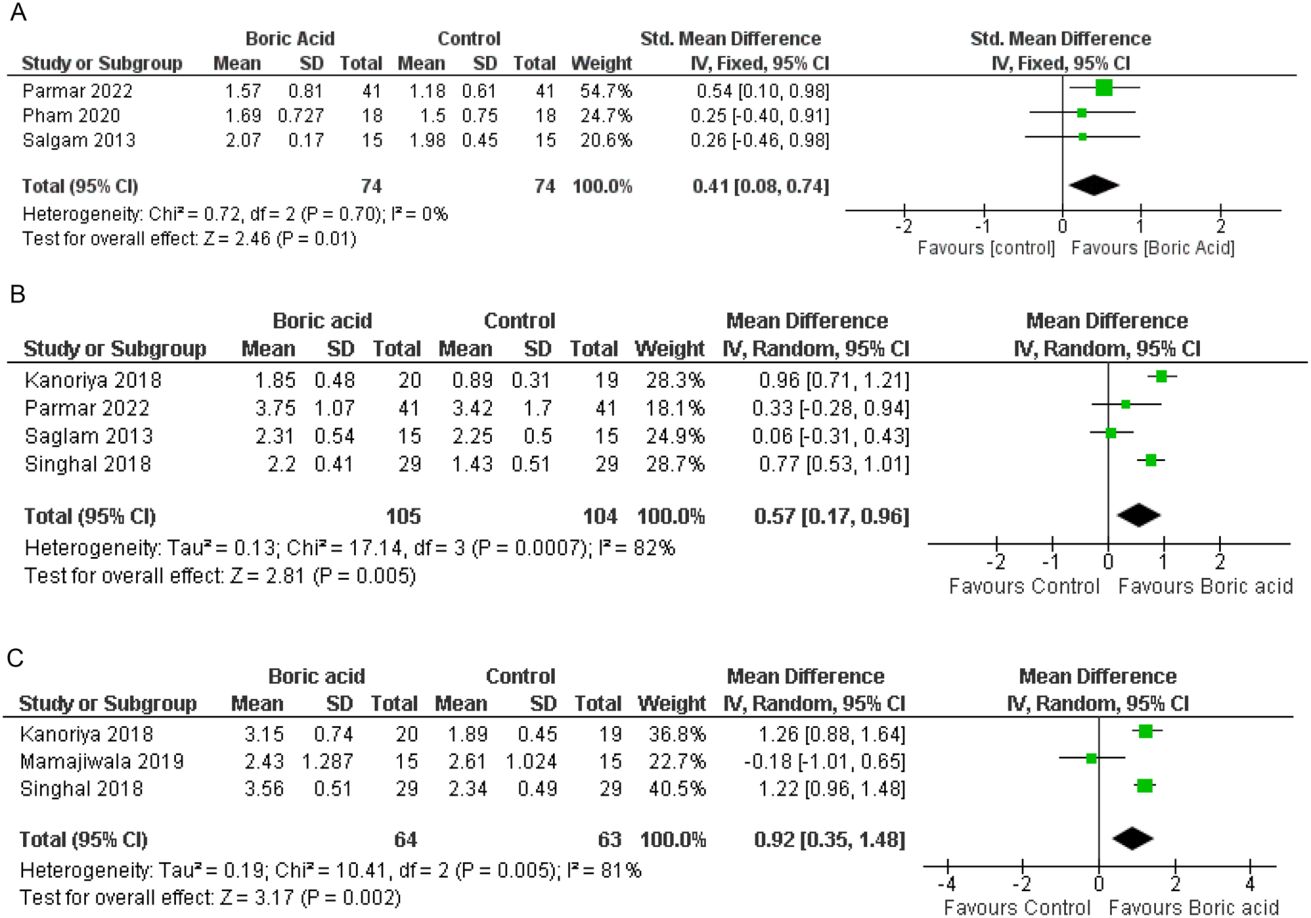
Forest plot of PPD reduction at **A**; 1 month follow-up, **B**; 3-month follow-up, **C**; 6-month follow-up

At the 1-month follow-up, the meta-analysis of 3 included studies [18, 19, 21], each comprising 74 participants per group, revealed a mean difference (MD) of 0.41 mm reduction in PPD in the boric acid group, with a 95% confidence interval (CI) of (0.08, 0.74) using the fixed-effects model to address heterogeneity (I^2^ = 0%). We found a statistically significant difference between the two groups (*P* = 0.01) (Fig. 3A).

At the 3-month follow-up, analysis of four studies [14, 15, 18, 19] involving 105 participants in the boric acid group and 104 participants in the control group indicated an MD of 0.57 mm reduction in PPD in the boric acid group, with a CI of (0.17, 0.96). Significantly, there was a notable difference between the groups, with greater improvement observed in the boric acid group compared to the control group (*P* = 0.005) Fig. 3B).

Moving to the 6-month follow-up, three studies [14, 15, 20] with a total of 64 participants in the boric acid group and 63 participants in the control group demonstrated an MD of 0.92 mm reduction in PPD in the boric acid group, with a CI of (0.35, 1.48). Importantly, a statistically significant difference was observed between the groups, with superior improvement noted in the boric acid group compared to the control group (*P* = 0.002) (Fig. 3C).

#### Clinical attachment level

**Fig. 4:**
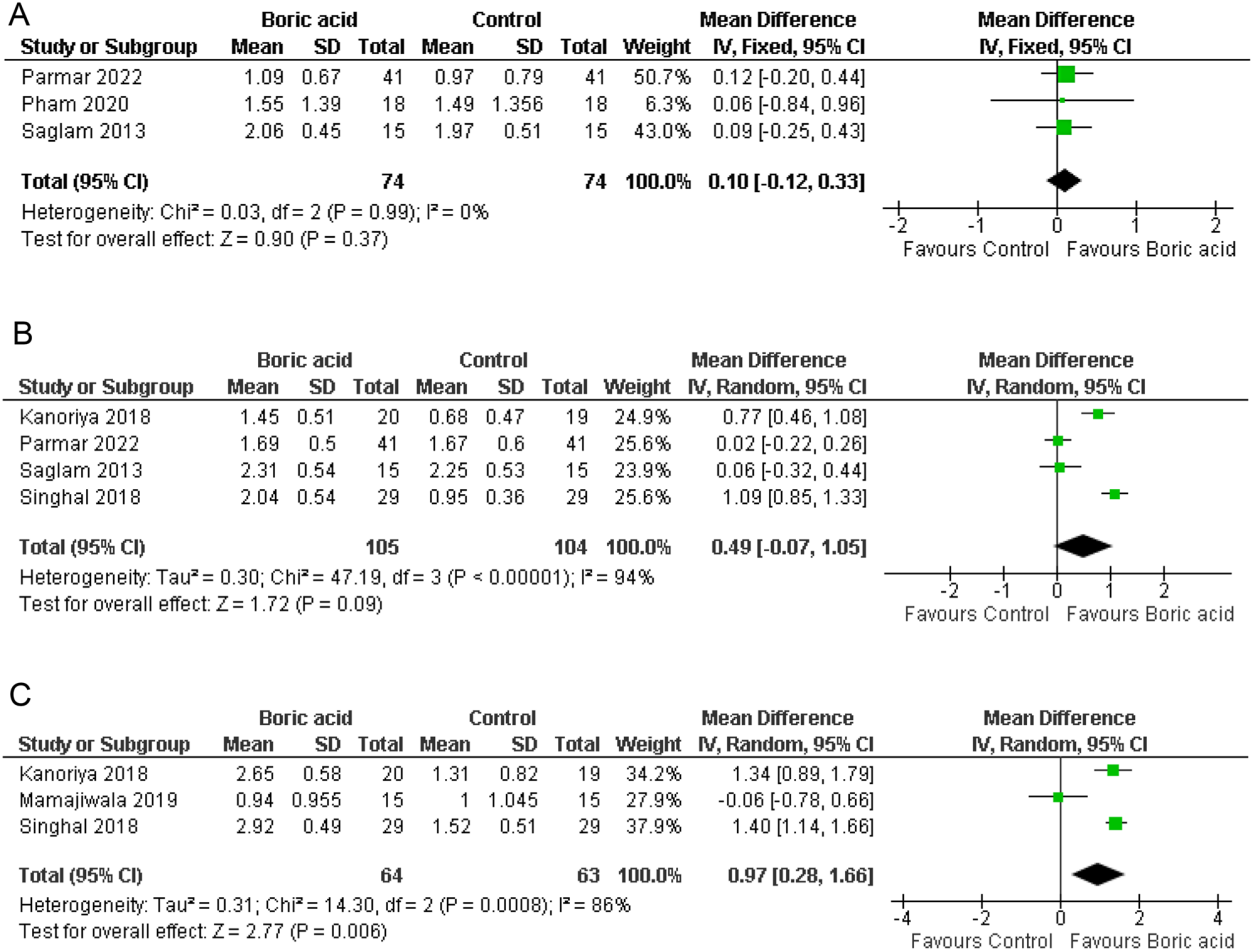
Forest plot of CAL at **A**; 1 month follow-up, **B**; 3-months follow-up, **C**; 6 months follow-up

At the 1-month follow-up, analysis of three studies [18, 19, 21], each comprising 74 participants per group, revealed a MD of 0.10 increase in CAL gain in the boric acid group, with a CI of (-0.12, 0.33). Nonetheless, no statistically significant difference was found between the two groups (*P* = 0.37) (refer to Fig. 4A).

Moving to the 3-month follow-up, examination of four studies [14, 15, 18, 19] involving 105 participants in the boric acid group and 104 participants in the control group demonstrated an increase in CAL gain in the boric acid group, with an MD of 0.49 and a CI of (-0.07, 1.05), with substantial heterogeneity (I^2^ = 94%). Despite this adjustment, there remained no statistically significant difference between the groups (*P* = 0.09) (Fig. 4B).

At the 6-month follow-up, three studies [14, 15, 20] comprising 64 participants in the boric acid group and 63 participants in the control group illustrated an increase in CAL gain in the boric acid group, with an MD of 0.97 and a CI of (0.28, 1.66). Notably, a statistically significant difference was observed, indicating superior CAL improvement in the boric acid group (*P* = 0.006) (Fig. 4C).

#### Gingival index

**Fig. 5:**
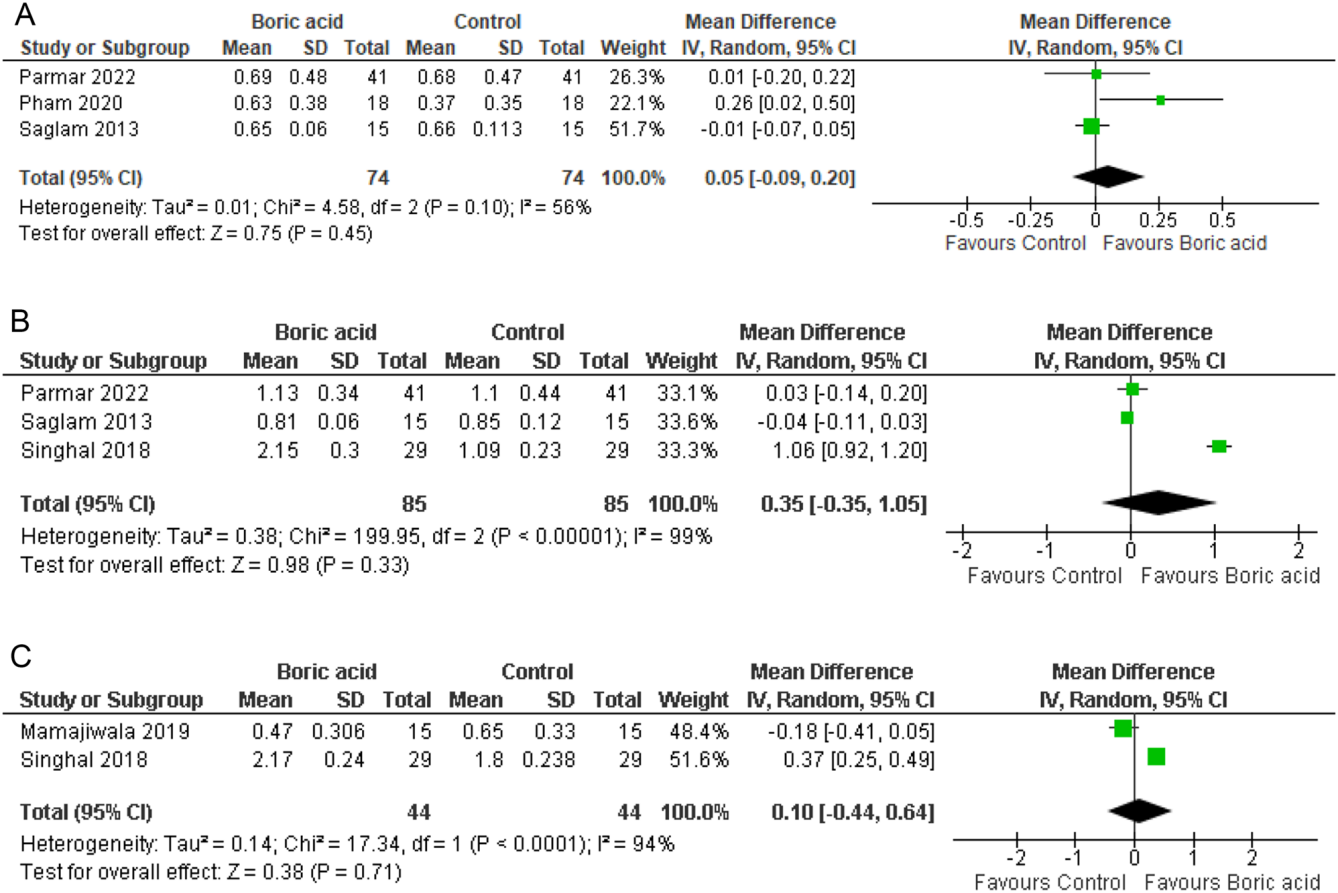
Forest plot of GI at **A**; 1 month follow-up, **B**; 3-month follow-up, **C**; 6-month follow-up

At the 1-month follow-up, analysis of four studies [18, 19, 21], each with 74 participants per group, indicated a MD of 0.05 in GI with a CI of (-0.09, 0.20) and moderate heterogeneity (I^2^ = 56%). Accordingly, the random-effects model was employed. However, no statistically significant difference was observed between the two groups (*p* = 0.45) (Fig. 5A).

At the 3-month follow-up, examination of three studies [14, 18, 19] involving 85 participants in both groups demonstrated an MD of 0.35 in GI with a CI of (-0.35, 1.05) and substantial heterogeneity (I^2^ = 99%), warranting the use of the random-effects model to address heterogeneity. Nevertheless, no statistically significant difference was found between the groups (*p* = 0.33) (Fig. 5B).

Moving to the 6-month follow-up, analysis of two studies [14, 20] comprising 44 participants in both groups revealed an MD of 0.10 in GI with a CI of (-0.44, 0.64) and notable heterogeneity (I^2^ = 94%), leading to the application of the random-effects model. Similarly, there was no statistically significant difference between the groups (*p* = 0.71) (Fig. 5C).

## Discussion

The current systematic review and meta-analysis aimed to comprehensively evaluate the efficacy of boric acid as a local drug delivery agent in periodontal diseases, with a focus on comparing its effects to a placebo. The varied follow-up durations, ranging from one to six months, facilitated the understanding of the temporal effects of boric acid treatment of periodontal parameters.

The comparative analysis of clinical outcomes, including PPD, CAL, and GI, revealed promising trends in favor of boric acid treatment. Significant improvements in periodontal parameters were observed across multiple studies. This systematic review included 6 Randomized clinical trials. Measuring the PPD change with time showed a significant reduction at 1, 3 and 6 months. CAL improvements were non-significant at 1 and 3 months but significant at 6 months. No significant differences in GI were noted at any follow-up.

The meta-analysis of PPD at different follow-up intervals revealed promising findings. At the one-month follow-up, there was a statistically significant difference between the boric acid and control groups, a trend towards reduction in PPD was observed in the boric acid group. In addition, at three and six months, significant reductions in PPD were noted in the boric acid groups compared to the control groups, indicating the potential efficacy of boric acid as an adjunctive therapy to SRP in improving periodontal health. In addition, analysis of CAL at different follow-up intervals demonstrated mixed findings. At the one-month and 3 months follow-up, no statistically significant difference was observed between the boric acid and control groups. However, at six months, significant improvements in CAL were noted in the boric acid groups compared to the control groups, suggesting a potential role of boric acid in promoting periodontal tissue attachment and stability over time.

The observed improvements in PPD and CAL gain in the boric acid groups may be attributed to the antimicrobial and anti-inflammatory properties of boric acid. Boric acid has been shown to exhibit bactericidal activity against periodontal pathogens, thereby reducing microbial load and inflammation within periodontal pockets. Additionally, boric acid may promote periodontal tissue healing and regeneration, leading to reductions in PPD and increase of CAL gains over time [13, 22]. Boric acid, a bioactive trace element, exhibits antibacterial effects and regulates inflammation and immunity [22]. Balci Yuce et al. demonstrated the ability of boric acid to reduce inflammation and bone loss in periodontitis [13]. Boric acid’s antibacterial and anti-inflammatory properties are attributed to AN0128, a boron-containing compound. AN0128 reduces tumor necrosis factor-α release and promotes osteogenic effects in human bone marrow stromal cells [23, 24]. 

The meta-analysis of GI at different follow-up intervals yielded inconclusive results. No statistically significant difference was observed between the boric acid and control groups at one, three, or six months, indicating that boric acid may have limited effects on gingival inflammation compared to SRP alone. However, it is essential to interpret these findings with caution due to the substantial heterogeneity observed across studies, which may influence the strength of the results.

Several hypotheses can explain the inconsistent effect of boric acid as a local drug on GI compared to PPD and CAL. First, the periodontal tissues may respond differently to the boric acid local drug. While boric acid is well known for its anti-inflammatory and antimicrobial properties, it may target deeper tissues improving the PPD and CAL. In contrast, the gingiva as superfacial tissue may be less affected by the boric acid local drug. Gingival inflammation is influenced by various factors, including plaque accumulation, host immune response, vitamin deficiency and systemic conditions, which may confound the effects of adjunctive therapies such as boric acid [25–29]. In addition, the lack of patient oral hygiene negatively impacts gingival health and may be the cause behind the lack of positive effect of boric acid on GI among the included studies. Finally, the assessment of gingival inflammation using the GI may be subjective and prone to inter-examiner variability, potentially masking subtle differences between treatment groups [28, 30–32]. 

### Comparison with the other published reviews

We found only one published review included 4 randomized controlled trials. The trials assessed the efficacy of boric acid as an adjunct to non-surgical periodontal therapy, either delivered as a gel or an irrigation. Results indicated that boric acid adjunctive therapy led to improvements in PPD and CAL at 3- and 6-months post-therapy, with statistically significant reductions in PPD and gains in CAL observed at the 6-month follow-up. Significant heterogeneity was noted in studies evaluating outcomes at 3 months, possibly due to differences in treatment protocols, particularly the mode of boric acid delivery.

However, in our systematic review we included 6 studies assessing the therapeutic potential of boric acid as a local drug delivery agent in periodontal diseases. In the follow up durations, we focused on short-term (one month), mid-term (3 months), and long-term treatment (6 months) effects. Comparative analysis of clinical outcomes indicated positive trends in PPD, CAL, and GI improvements in the boric acid groups compared to controls at different follow-up periods. While the published review lacks the meta-analysis of GI, our Meta-analysis results for GI demonstrated no statistically significant difference between boric acid adjunctive therapy and control groups at 1, 3, and 6-months follow-up.

It is crucial to limit the generalizability of our findings in this systematic review as we only included 6 eligible studies. We acknowledge the variability among studies in the methodology, formula of boric acid, control groups, and the studied population. The small sample size, combined with variations within the formulations, and dosages, as well as the heterogeneity in baseline characteristics of the members, limits the external validity of our results. Therefore, even as boric acid indicates promise as local drug delivery for periodontitis, the conclusions drawn from this evaluation have to be seen as preliminary. Larger, properly designed research with standardized protocols is essential to verify the efficacy of boric acid and enhance broader applicability in clinical exercise.

## Clinical implications

The findings of this systematic review have significant clinical implications for periodontal therapy. Boric acid, as a local drug delivery agent, offers a potential adjunctive treatment option to enhance the efficacy of non-surgical periodontal therapy. Clinicians may consider incorporating boric acid into their treatment protocols for patients with periodontal diseases, particularly those demonstrating inadequate responses to conventional therapy or at increased risk of disease progression. Furthermore, the relatively low cost and favorable safety profile of boric acid enhance its attractiveness as a therapeutic option in resource-limited settings.

## Limitations

Despite the overall positive findings, certain limitations of the included studies require attention. Variability in study designs, patient populations, and outcome measures may have introduced heterogeneity into the analysis, potentially influencing the strength of the results. Additionally, the limited number of studies included in the meta-analysis may have restricted the generalizability of the findings. Furthermore, Lack of standardized formulations of boric acid optimized for dental applications, making it more challenging to ensure consistent and effective treatment outcomes. The inclusion of research with a high risk of bias is a limitation of this review. While these studies have been retained to provide a complete synthesis of evidence, we acknowledge that their methodological limitations may affect the reliability of the findings. Future research should prioritize rigorous study designs to decrease bias and improve the quality of evidence.

In conclusion, the systematic review and meta-analysis presented compelling evidence supporting the therapeutic potential of boric acid as a local drug delivery agent in periodontal diseases. By synthesizing existing evidence and providing quantitative insights, the study contributes valuable knowledge to the field of periodontology, informing clinical practice and guiding future research endeavors. Further investigations with standard protocol for boric acid formulations and application are warranted to elucidate the mechanisms underlying boric acid’s effects on periodontal tissues and to optimize its clinical application in the management of periodontal diseases.

## Electronic supplementary material

Below is the link to the electronic supplementary material.

Supplementary Material 1

Supplementary Material 2

## Data Availability

The data that support the findings of this study are available from the corresponding author upon reasonable request.
