# Supplementary material for: Therapeutic potential of boric acid as a local drug delivery agent in periodontitis: a comprehensive systematic review and meta-analysis
Source: BMC Oral Health. 2025 Jan 17;25:88. doi: 10.1186/s12903-025-05445-0 (PMC11740524; doi:10.1186/s12903-025-05445-0)
Supplement: Supplementary file 1 — Supplementary Material 1 [file 12903_2025_5445_MOESM1_ESM.docx]

**Supplementary File 1**

**Search terms for each database**

- **Cochrane Central Register of Controlled Trials:**
  **Search terms:** "boric acid" OR "local drug" OR "subgingival irrigation" AND "periodontitis" OR "periodontal pocket"
  **Filters applied**: Clinical trials only.
- **MEDLINE-PubMed:**
  **Search terms:** ("boric acid"[MeSH Terms] OR "local application") AND ("periodontal disease" OR "periodontal pocket").
  **Filters applied:** None.
- **EMBASE:**
  **Search terms:** 'boric acid' OR 'topical application' OR 'subgingival irrigation' AND 'SRP' OR 'periodontal disease'.
- **Web of Science:**
  **Search terms:** "boric acid" AND ("periodontitis" OR "local drug delivery" OR "topical application").
  **Filters applied:** None.
- **Scopus:**
  Search terms: "boric acid" AND ("local application" OR "subgingival irrigation") AND ("periodontal disease" OR "periodontal pocket").
