# Supplementary material for: Therapeutic potential of boric acid as a local drug delivery agent in periodontitis: a comprehensive systematic review and meta-analysis
Source: BMC Oral Health. 2025 Jan 17;25:88. doi: 10.1186/s12903-025-05445-0 (PMC11740524; doi:10.1186/s12903-025-05445-0)
Supplement: Supplementary file 2 — Supplementary Material 2 [file 12903_2025_5445_MOESM2_ESM.docx]

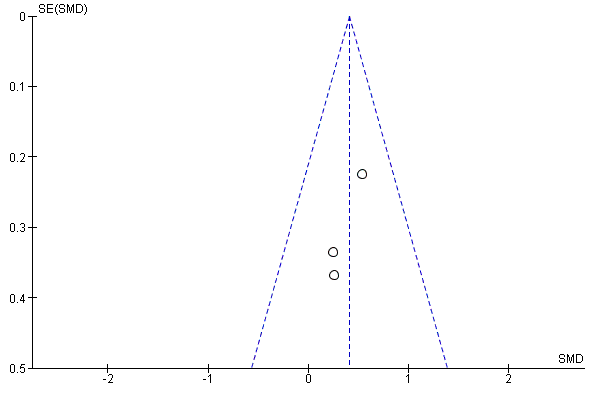


Funnel plot of comparison: 1 PD reduction at 1 month follow-up, outcome: 1.1 PD reduction.


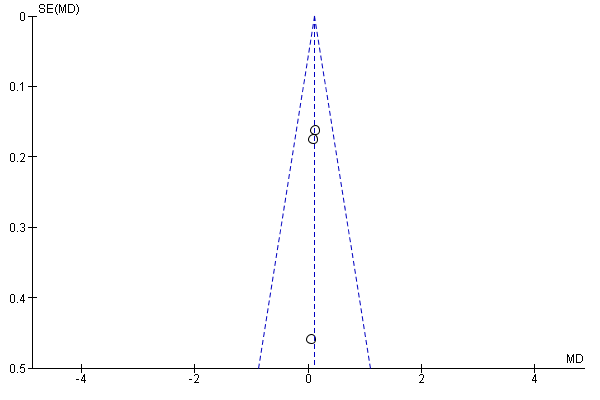


Funnel plot of comparison: 2 CAL gain at 1 month follow-up, outcome: 2.1 CAL gain.


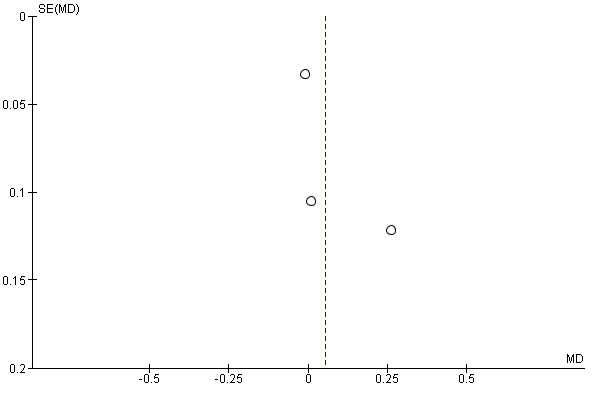


Funnel plot of comparison: 3 GI at 1 month follow-up, outcome: 3.1 GI.


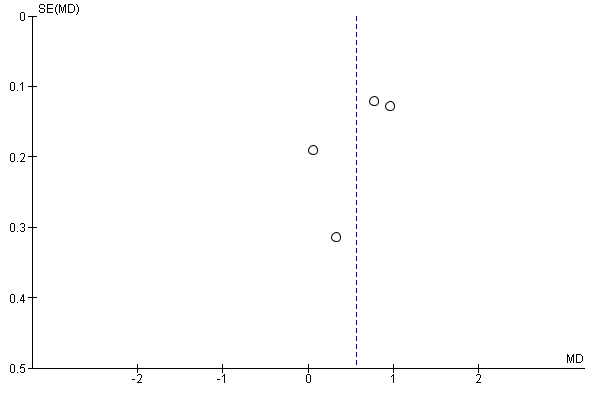


Funnel plot of comparison: 4 PD reduction at 3 month follow-up, outcome: 4.1 PD reduction.


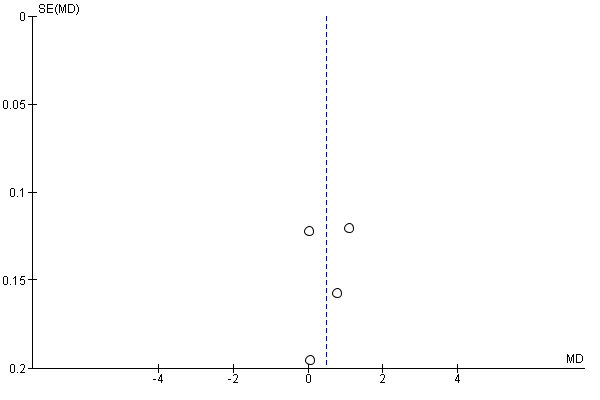


Funnel plot of comparison: 5 CAL gain at 3 month follow-up, outcome: 5.1 CAL gain.


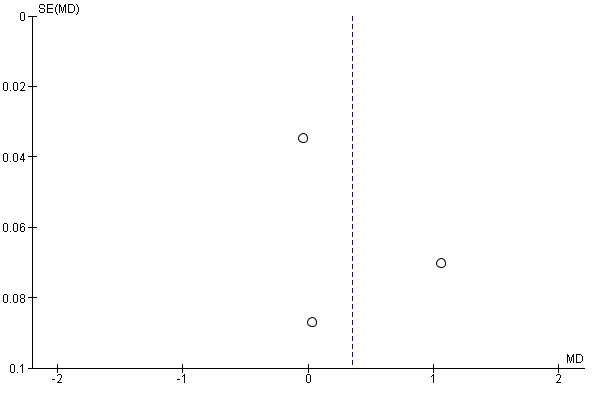


Funnel plot of comparison: 6 GI at 3 month follow-up, outcome: 6.1 GI.


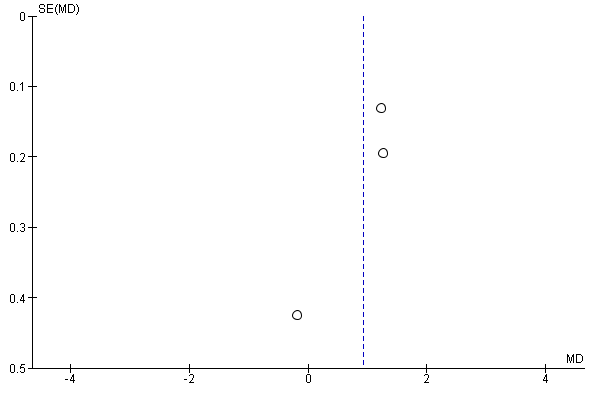


Funnel plot of comparison: 7 PD reduction at 6 month follow-up, outcome: 7.1 PD reduction.


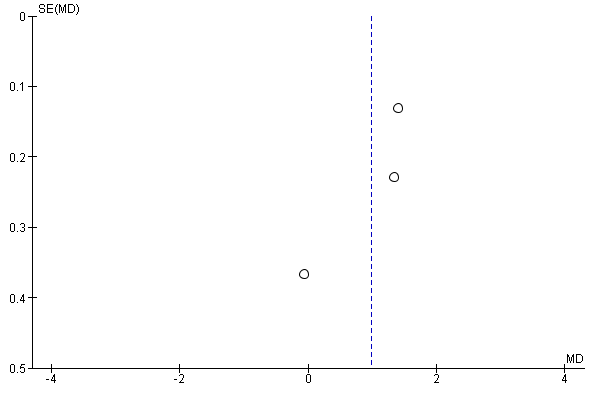


Funnel plot of comparison: 8 CAL gain at 6 month follow-up, outcome: 8.1 CAL gain.


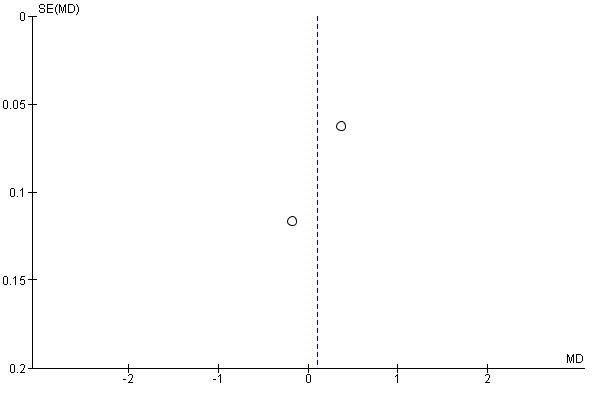


Funnel plot of comparison: 9 GI at 6 month follow-up, outcome: 9.1 GI.
